# Cointegration Approach for Vibration-Based Misalignment Detection in Rotating Machinery Under Varying Load Conditions

**DOI:** 10.3390/s25216764

**Published:** 2025-11-05

**Authors:** Sylwester Szewczyk, Roman Barczewski, Wiesław J. Staszewski, Damian Janiga, Phong B. Dao

**Affiliations:** 1 AGH University of Krakow, Faculty of Mechanical Engineering and Robotics, Department of Robotics and Mechatronics, al. Mickiewicza 30, 30-059 Krakow, Poland; sszewczyk@agh.edu.pl (S.S.); staszews@agh.edu.pl (W.J.S.); 2 ABB Business Services sp. z o.o., Process Automation Marine & Ports, Starowiślna 13a, 31-038 Krakow, Poland; janiga@agh.edu.pl; 3 Poznan University of Technology, Faculty of Mechanical Engineering, Institute of Applied Mechanics, ul. Jana Pawła II 24, 60-965 Poznan, Poland; roman.barczewski@put.poznan.pl; 4 AGH University of Krakow, Faculty of Drilling, Oil and Gas, Department of Petroleum Engineering, al. Mickiewicza 30, 30-059 Krakow, Poland

**Keywords:** rotating machinery, misalignment detection, vibration measurements, varying load effects, cointegration, Augmented Dickey–Fuller (ADF) test, condition-based maintenance, data analysis

## Abstract

Shaft misalignment is among the most common faults in rotating machinery, and although many diagnostic methods have been proposed, reliably detecting it under varying load conditions remains a major challenge for vibration-based techniques. To address this issue, this study proposes a new vibration-based misalignment detection framework that leverages cointegration analysis. The approach examines both the stationarity of vibration signals and the residuals derived from the cointegration process. Specifically, it combines the Augmented Dickey–Fuller (ADF) test with cointegration analysis in three stages: (1) applying the ADF test to raw vibration data before cointegration, (2) performing cointegration on the vibration time series, and (3) reapplying the ADF test to the post-cointegrated data. The method was validated using experimental measurements collected from a laboratory-scale test rig comprising a motor, gearbox, and hydraulic gear pump, tested under both healthy and misaligned states with varying degrees of severity. Vibration signals were recorded across multiple load conditions. The results demonstrate that the proposed method can successfully detect misalignment despite load variations, while also providing insights into fault severity. In addition, the residuals from the cointegration process proved to be highly sensitive to damage, highlighting their value as features for vibration-based condition monitoring.

## 1. Introduction

Many rotor–shaft systems are critical concerning operation and have a high capital cost. Turbogenerators in power stations or marine propulsion systems in ships are good examples. Predictive maintenance and condition monitoring of such machines is vital. Monitoring for excessive levels of vibration that can lead to rotor, gearbox, or bearing failures is of particular importance. Vibration in rotating machinery is often caused by improperly balanced and/or aligned shafts due to manufacturing or operation. Alignment of shafts and couplings in a collinear line is important for reliable operation of rotor–shaft systems. Any deviation from this position with respect to axis rotation known as misalignment is one of the most common faults in rotating machinery. There are three different types of misalignments, i.e., parallel, angular, and combined. Since perfect alignment is not possible in practice, monitoring for misalignment is important to prevent failures of rotors, couplings (rigid, gear, or flexible), bearings, seals, lubricant leakage, or eventually structure collapse. Various condition monitoring methods have been developed for misalignment detection and monitoring. These include shock pulse measurement [1], acoustic emission [2], temperature measurement/imaging [3,4], laser-based metrology [5,6,7], torque analysis [8], motor current monitoring [9,10,11], oil analysis [12], acoustic measurements [13,14], and vibration analysis [12,15,16].

Vibration measurements are particularly attractive for misalignment detection due to limited costs and sensitivity to small faults. Many operating rotating machines have been already equipped with permanently attached accelerometers that are part of condition monitoring systems. Therefore, many different vibration-based approaches have been developed in this area. Altogether, these approaches can be classified into feature-based methods and knowledge- or expert-based methods. The former employs vibration spectra analysis [17], instantaneous frequency estimation [18], principal component analysis [19], wavelet analysis [20,21], dynamic stability analysis [22], and singularity analysis [23]. The latter includes model-based approaches [16,24], object-oriented programming [25], and neural networks [26]. Combined signal- and model-based approaches have also been used for misalignment detection [27]. A good overview of various misalignment detection methods is given in [28,29].

Vibration analysis is by far the most widely used approach for misalignment detection. However, previous research studies show that speed, coupling type, and load have a strong effect on vibration measurements and spectra [28,30]. When vibration-based features are used for fault detection in rotating machinery, different faults often exhibit similar features. This is particularly relevant to misalignment detection. Many vibration-based features used for misalignment are not unique, as indicated in [31]. Excessive amplitude levels of the second harmonic—often used for misalignment detection—may indicate not only misalignment but also nonlinearities related to material- or fluid-based failures. In addition, overloaded (e.g., due to frequent start-ups) and unloaded or lightly loaded roto-shaft systems with gearboxes lead to transient vibration that is very difficult to analyze. The latter scenario often involves rattling, which adds significant background noise to the recorded data. Knowledge-based methods and soft computing approaches could offer a solution to this problem. However, both approaches are computationally expensive. Knowledge-based methods often require accurate models, which are difficult to obtain, and the latter requires significant amounts of data that are not always desired and/or available. Various methods based on time, frequency, and combined time–frequency approaches have been developed for condition monitoring, as summarized in [32]. Analysis of non-stationarity in vibration measurements—which can uniquely identify possible machine faults—is another solution to the problem. Cointegration analysis that relates to stationarity and non-stationarity of data is one of the interesting developments in this area. The method originates from econometrics [33]. It has been brought to engineering for process control monitoring [34] and then has been mainly used for damage detection in structural health monitoring [35,36,37,38,39,40,41,42]. The method allows one to remove common trends that exist in the analyzed data due to operational and environmental conditions. Once this is achieved, cointegration residuals are analyzed for structural damage detection. Recently, cointegration has been combined with other statistical analysis methods and machine learning (ML) algorithms to improve damage detection in large-scale structures [43,44,45,46,47]. Condition monitoring applications of this approach are still relatively limited and include methods for fault detection in gearboxes and ball-bearings [48,49]. Cointegration has been successfully applied for condition monitoring of wind turbine systems [50,51,52,53,54] and for damage assessment of offshore platforms [55]. A recent overview of various developments related to cointegration-based damage detection in structures and fault detection in condition monitoring is given in [56].

This paper proposes a misalignment detection method based on cointegration and unit root tests. The presented techniques are able to handle vibration data, remove the effect caused by varying load conditions, detect misalignment, and classify its severity. The work presented in this paper utilizes cointegrating residuals that are formed by projecting vibration data on the cointegration vector. Additionally, the Augmented Dickey–Fuller (ADF) test [57], applied on both raw vibration data and cointegration residuals, is used not only for the purpose of testing the degree of stationarity but also to develop a misalignment detection indicator.

The main contributions of this paper are summarized as follows:

- A misalignment detection method for rotor–shaft systems is proposed, based on cointegration theory and unit root tests.
- The proposed technique is capable of processing vibration data under varying load conditions, effectively removing the influence of operational variations.
- The method enables both detection and severity classification of misalignment faults through the analysis of cointegrating residuals.
- The ADF test is applied to both raw vibration data and cointegration residuals, providing a robust misalignment detection indicator.

It is important to note that ML–based fault diagnosis has become a major research focus in condition monitoring of rotating machinery. ML offers clear advantages over traditional approaches, including the ability to process complex data, automate analysis, adapt to diverse fault types, and improve performance through continuous learning. Despite these benefits, ML-based methods face key challenges such as dependence on high-quality labeled data, limited interpretability, and integration complexity. Recent reviews [58,59] highlight the rapid rise of supervised and unsupervised algorithms for intelligent fault detection within Industry 4.0 frameworks. Although these methods often achieve high prediction accuracy, their reliance on extensive training data and black-box behavior remains a limitation. In contrast, the present study introduces a statistically grounded approach based on cointegration analysis, providing a transparent and data-efficient solution for fault detection without requiring large-scale training datasets.

The structure of this paper is as follows. The cointegration analysis is briefly introduced in Section 2, starting from the concept of stationarity. The algorithm proposed for misalignment detection is introduced in Section 3. The algorithm is based on cointegration theory and unit root testing. Experiments undertaken to validate the method are described in Section 4. A simple rotor-based system with parallel misalignment is used in these investigations. Section 5 presents the details of the cointegration-based approach proposed in this paper. Section 6 presents the experimental results and discussion. Finally, conclusions are described in Section 7, giving the pros and cons of the proposed approach and proposing possible future research in the field.

## 2. Cointegration—Theoretical Background

For the sake of completeness, this section briefly introduces the concept of cointegration needed to introduce the fault detection method proposed in this paper. Since cointegration analysis relates to data stationarity, basic definitions of stationarity and non-stationarity are given firstly to highlight some differences between engineering, mathematics, or time series analysis. The strict stationarity defined in mathematics or weak stationarity commonly known in engineering is not sufficient to understand the concept of cointegration. Then, cointegration and relevant statistical tests are only briefly introduced, with references for more details and further reading.

### 2.1. Stationarity and Non-Stationarity

In mathematics and statistics, stationary processes—represented by time series $\left\{X_{t}\right\}\;$ of observations given for time *t*—relate to stochastic processes whose joint unconditional probabilities *D* do not change over time τ, i.e.,

(1) $$D(\left\{X_{t}\right\})=D(\left\{\;X_{t+\tau }\right\})\;$$

Consequently, for equally spaced samples, weak stationarity of time series in engineering applications implies that its mean is constant, the auto-covariance does not vary over time, and its variance is finite for all times [60], i.e.,

(2) $$\;\;\;E\left[\left\{X_{t}\right\}\right]=E\left[\left\{X_{t+\tau }\right\}\right]\;Cov\left(t_{1},t_{2}\right)=Cov\left(t_{1}-t_{2},0\right)\;E\left[{\left|\left\{X_{t}\right\}\right|}^{2}\right]<\infty$$

Time series analysis in econometrics and statistics uses time-based trend models to explain the difference between stationarity and non-stationarity. This is often based on autoregressive processes of first order, given as follows:

(3) $$x_{t}=\;\alpha +\phi x_{t-1}+\;\varepsilon_{t},$$

where *ε*_*t*_ describes an independent process of zero mean Gaussian noise and ϕ is a coefficient. The above equation shows that the time series is regressed on previous values. This coefficient defines three types of time series [60,61]: (1) stationary processes that exhibit jagged edges and fluctuate around mean values ($\alpha =0,\;\;\left|\phi \right|<1$); (2) non-stationary processes that appear finer and gradually intensify ($\alpha =0,\;\;\left|\phi \right|>1$) until diverging drastically; and (3) pure random walks that involve alternating upward and downward movements, resembling non-stationary patterns but occurring at slower paces (α=0,  ϕ=1). For α≠0, random walk with a drift is obtained.

Stationarity of data can be violated by sudden changes (e.g., impulses) and/or trends. For example, in rotating machinery, impulses are often caused by faulty teeth in gearboxes or faulty rings in ball-bearings, and trends result from operational and/or environmental conditions. In engineering applications, it is important to distinguish between non-stationary behavior and trends. Trend-stationary processes can be described as follows:

(4) $$x_{t}=\;\gamma_{0}+\gamma_{1}t+e_{t}$$

whereas difference-stationary processes can be defined by

(5) $$x_{t}=\alpha_{0}+x_{t-1}+e_{t}$$

where *e_t_* is a stationary process and $\gamma_{0}$, $\gamma_{1}$, $\alpha_{0}$ are coefficients.

### 2.2. Unit Roots and Testing for Unit Roots

Non-stationary data are often transformed into stationary data in time series analysis. A series of successive differences—which can be denoted as *I*(*d*)—can transform non-stationary time series into stationary time series. Non-stationary time series that exhibit stationary after *d* differences are called integrated time series of order *d*. *I*(0) is a non-integrated (i.e., stationary without a trend) time series, and *I*(1) is a process—called a random walk—that needs to be differenced once to become *I*(0) process. Thus, differencing is just subtracting *x*_*t*−1_ from *x_t_*. As a result, the analyzed process loses one observation.

Generally, time series *X_t_* is *I*(*d*) if the following process is stationary without a trend (where *L* is a lag operator):

(6) $$z_{t}={\left(1-L\right)}^{d}X_{t}$$

In time series analysis, unit root processes are often used interchangeably with non-stationary processes. However, these two processes are different. A unit root process contains a random walk process and is described as *I*(1) process. All unit root processes are non-stationary, but not vice versa. Therefore, detrending and time-differencing processes are different. In contrast to first time-differencing, detrending of unit root processes does not result in stationarity.

Two prerequisites are required to conduct the cointegration test [60,61]: Firstly, examined time series must possess identical levels of non-stationarity (or the order of integration). Secondly, they should exhibit mutual trends. Thus, checking for unit roots is important to establish the level of stationarity. The ADF test [57] is often used to achieve this task. This test formulates the null and the alternative hypotheses. The former is that there is a unit root in the time series, whereas the latter is that the analyzed time series is stationary. The relevant ADF equation that is analyzed can be expressed as follows:

(7) $$∆x_{t}=\alpha +\phi x_{t-1}+\sum_{i=1}^{p}\beta_{i}∆x_{t-i}+\gamma t+\varepsilon_{t},$$

where *p* is the lag length, and α and γ are a constant and a trend, respectively. The regression function given in Equation (7) allows for “no-constant”, “constant only”, and “constant and trend” scenarios, depending on the values of the relevant coefficient. The crucial point is to capture the inherent trend characteristics present in the data effectively. The ADF unit root test involves the t-statistic, which are pivotal in the entire procedure and in deciding whether to accept/reject the tested hypotheses. If these statistics are larger than the critical threshold, the null hypothesis is rejected (no unit root present; stationarity *I*(0)). Otherwise, the null hypothesis remains unchallenged (unit root present; non-stationarity *I*(1)).

Equation (7) shows that the ADF test involves the estimation of the *p* lag length. This parameter decides the number of periods involved and is crucial for the entire cointegration analysis. The balance between the lag length being too limited or excessively prolonged is important. Several methods have been developed to achieve this task, as discussed in [61]. The ADF t-statistics are used to establish the lag length in this paper. The t-statistics are used to determine the level of stationarity in the ADF tests. More negative statistics indicate more stationarity [56,61]. Since potential fault/damage has an inevitable influence on data stationarity, the t-statistics are also used to establish the lag length in the ADF test. The process involves four steps. Firstly, the minimum and maximum values of lag length are established. For *N* data samples, the former is equal to $p_{min}$ = 1, whereas the latter can be calculated as follows [62]:

(8) $$p_{max}=12{\left(\frac{N}{100}\right)}^{1/4}$$

Secondly, cointegration analysis described in Section 2.3 is performed for all possible lag lengths. Then, ADF t-statistics are calculated for all lag lengths and all cointegration residuals. Finally, the lag length that produces the most negative value of the t-statistic is selected. More details about this procedure can be found in [61].

### 2.3. Cointegration

The non-stationary-to-stationary transformation and differencing is the purpose of cointegration. The method is used to establish whether there is a correlation between different time series in the long term. The concept of cointegration was introduced in econometrics to analyze various trends and relationships between economic variables (e.g., currencies, shares, commodities) [60].

Assuming that time series *X*_1_, *X*_2_, *… X_n_* are non-stationary and integrated, cointegration is a statistical property of these time series variables. These non-stationary time series are cointegrated if their linear combination has a lower order of integration and finally becomes a stationary time series, i.e.,

(9) $$\beta X=\beta_{1}X_{1\;}+\beta_{2}X_{2\;}+\beta_{3}X_{3\;}+\cdots +\beta_{n}X_{n\;}\;$$

is *I*(0). The β vector in the above equation—called the cointegrating vector—dictates how these series are combined. It is important to note that this vector is not unique, i.e., times series can be cointegrated in different ways. This vector can be normalized as follows to establish a unique identification of *β*:

(10) $$\beta =(1,\;-\beta_{2\;},\;-\beta_{3}\;,\ldots ,-\beta_{n})$$

Then, Equation (9) can be re-written using a standard regression form as follows:

(11) $$X_{1}=\;\beta_{2}X_{2}+\;\beta_{3}X_{3}+\cdots +\;\beta_{n}X_{n}+\;u_{t},$$

where $u_{t}$ is a stationary correcting error that intuitively describes how far locally the new process deviates from a long-term equilibrium. This error exhibits stationary *I*(0) property, and for a state of long-term equilibrium, it tends to zero, so the variables involved are in balance. The stationary correcting error is often called the cointegration residual. Cointegration implies that involved time series are connected through an error correction model that allows one to understand the long-run dynamics.

For a multi-variate cointegrated system and cointegrated vector described by Equation (9), the Vector Error Correction Model (VECM) is a function of deviations from the long-run equilibrium. If the cointegrated vector is known, the VECM for bivariate systems can be obtained using ordinary least squares estimators or maximum likelihood estimation. However, for multi-variate systems, special tests are needed. The Johansen’s cointegration test [63] is used to determine the number of cointegrated vectors and to obtain the VECM using maximum likelihood. The Johansen’s cointegration test—based on the maximum likelihood technique—establishes whether the cointegration is present and determines the count of relationships that cointegrate. The procedure follows the Vector Auto-Regressive (VAR) model of order *k*, which can be described as follows:

(12) $$X_{t}=\;\Phi D_{t}+\;\Pi_{1}X_{t-1}+\;\Pi_{2}X_{t-2}+\cdots +\;\Pi_{k}X_{t-k}\;+\;\varepsilon_{t},$$

where *X_t_* is a vector of data, $\Pi_{i}$ is a matrix of parameters, $\varepsilon_{t}$ is the normally distributed serially uncorrelated error, and $\Phi D_{t}$ represents the deterministic trend. Following Equations (9)–(11), Equation (12) can be modified to become the VECM:

(13) $$X_{t}=\;\Phi D_{t}+\;\Pi_{1}\Upsilon_{t-1}+\;\Gamma_{1}{\Delta \Upsilon }_{t-1}+\cdots +\Gamma_{p-1}{\Delta \Upsilon }_{t-p+1}+\;\varepsilon_{t},$$

where Π and Γ are the matrices of long- and short-run impacts in the model. Within the VECM framework, both Δ*Υ* and its lagged values are integrated of order *I*(0). 

These sequential steps are used in the cointegration analysis:

- For *X_t_*, the VAR model given by Equation (12) is built.
- Statistical tests for likelihood ratio are conducted to assess the rank of matrix *Π*. The rank identifies linearly cointegrating relationships that are independent, leading to cointegration vectors.
- Normalization is used, if needed.
- Employing (normalized) cointegration vectors, cointegration residuals are calculated using the specified projection method.
- The maximum likelihood method is used to estimate a collection of error correcting variables for the cointegrated VECM (Equation (13)).

Since the scope of this paper is not sufficient to fully describe the entire cointegration procedure, further reading (e.g., [33,35,56,60,61,63]) is recommended for more details. In econometrics, the cointegration procedure is used to establish long-term correlations between time series and analyze possible trends and links between economic variables. In contrast, the method used in structural health and condition monitoring looks for possible data trends that relate to operational and/or environmental conditions, assuming that non-stationarity left in the data after cointegration relates indirectly to damage or faults. Previous research studies have mainly relied on ADF t-statistics and correlation residuals for this task.

### 2.4. Fractal Signal

Stationary periodic series remain unchanged when subjected to shifts in the time domain. Consequently, their statistical properties are also unaltered by translations. On the contrary, self-similar signals (often called 1/*f* signals, e.g., Gaussian noise or Brownian motion) do not resemble translations but maintain a consistent pattern across different scales [64]. Fractal signal processing, which involves time-scale analysis of statistically self-similar signals, is not only used to obtain fractal dimensions but also to extract features related to possible faults in rotating machinery, as explained in [64]. This is particularly relevant to vibration gearbox or ball-bearing data that is heavily corrupted by background noise.

The spectra of self-similar signals exhibit a specific pattern that can be described by the following formula:

(14) $$S_{x\;}\left(\omega \right)=\;\frac{\sigma_{x}^{2}}{{\left|\omega \right|}^{\gamma }},$$

where γ is the spectral parameter. Processes where 1 < *γ* < 3 display finite power with low considered frequency and are termed fractional Brownian motions. A specific instance of this is traditional Brownian motion, where *γ* = 2. Fractional Gaussian noises are processes with −1 < *γ* < 1 that have high-frequency finite power.

## 3. Misalignment Detection Methodology

This section introduces the cointegration-based method proposed for misalignment detection in a loaded rotor–shaft system. Vibration responses in such systems are heavily corrupted by noise. Any features related to faults are embedded in this background noise and are difficult to detect. Environmental and operational variability of vibration data used for damage/fault detection is often manifested by long- and short-term trends, as explained in [56]. The former is more common for structural vibration (long-term trends related to environmental conditions), whereas the latter could be related to rotating machinery vibration. Both types of trends are structure/machine-dependent.

**Figure 1:**
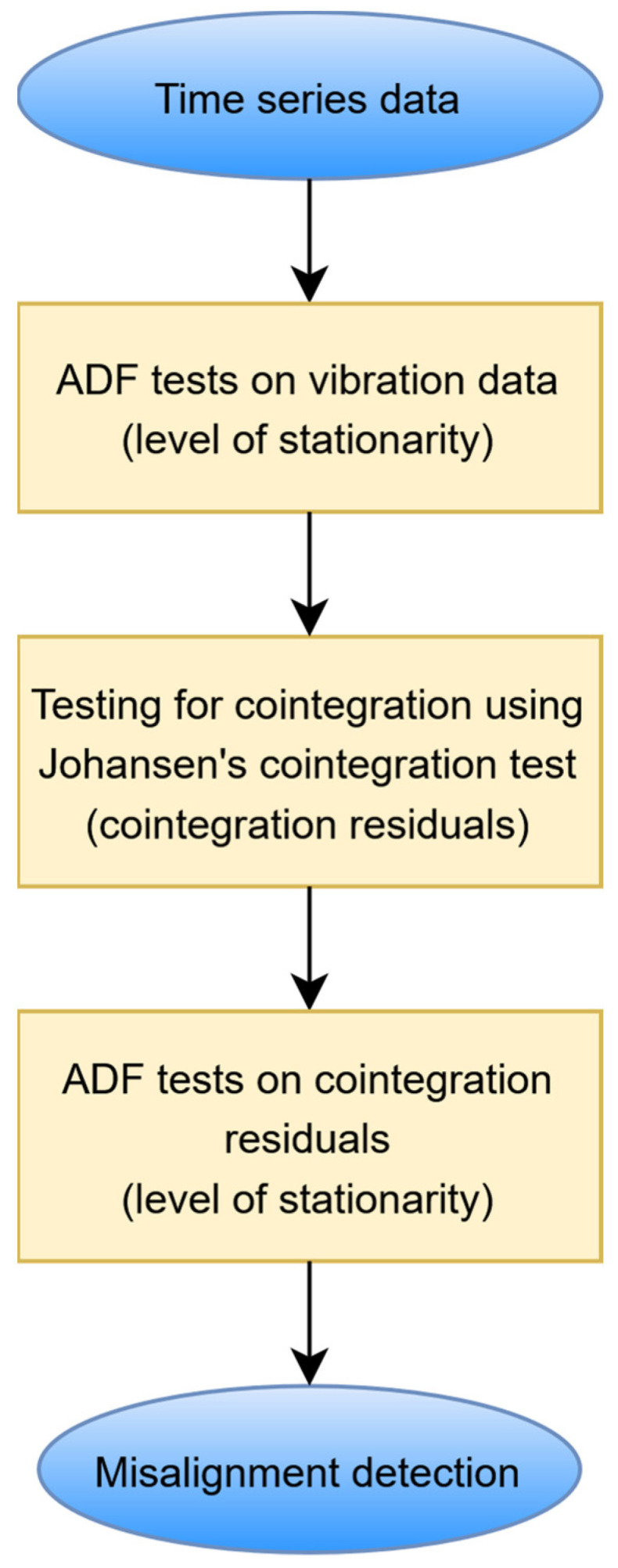
Schematic diagram illustrating the procedure used for misalignment detection.

There are two important questions here: How can undesired trends be removed from the data, and how can misalignment be detected using cointegration residuals? The assumption is that cointegration residuals are free from undesired trends when cointegration theory is applied. Then, the features of cointegration residuals can be used for fault detection. The proposed contemporary data processing procedure—based on recent developments in interdisciplinary stationarity analysis (intersection of mathematics, econometrics, and signal processing), illustrated in Figure 1—involves several steps that can be described as follows:

1. Data segmentation

The first step involves data segmentation. Vibration rotor—shaft responses—representing different fault conditions—are divided into 10 evenly distributed time series, which consist of the same number of data samples.

2. ADF tests for unit roots on vibration data

The ADF testdescribed in Section 2.2 is applied to vibration time series to establish the level of stationarity of these vibration time series.

3. Johansen’s cointegration test

The Johansen’s cointegration proceduredescribed in Section 2.3 is used to determine the number of cointegrated vectors and to obtain the VECM using maximum likelihood. Normalized cointegrating vectors are used to form cointegration residuals.

4. ADF tests for unit roots on cointegration residuals

The ADF testdescribed in Section 2.2 is applied to cointegration residuals to establish the level of stationarity of the cointegration residuals.

Figure 1 presents a schematic diagram illustrating the proposed procedure for misalignment detection. To complement this visual representation, the following pseudo-code, based on original MATLAB code, provides a detailed, stepwise description of the computational workflow. This Algorithm 1 outlines the data processing, statistical testing, and cointegration analysis stages that form the foundation of the method, enabling reproducibility and implementation clarity.
**Algorithm 1**. Cointegration-Based Misalignment Detection**Input**: Vibration response datasets {*L000, L025, L050, L075, L100*}**Output**: ADF t-statistics, cointegration residuals, and statistical stationarity1: **for** each condition *L* in {*L000, L025, L050, L075, L100*} **do**2:    Load vibration response data corresponding to condition *L*3:    Assemble 10 measurement segments into a time-series matrix4: **end for**5: Set number of lags nlag = 15 and deterministic term cterm = 0
6: Determine number of variables N = rank(data)
7: **for** each dataset *D* in {*L000, L025, L050, L075, L100*} **do**8:    **for** each signal i = 1 to *N* **do**9:     Perform ADF test on signal i of *D*10:   Record corresponding ADF t-statistic11:  **end for**12:  Apply Johansen cointegration test on signal D with parameters (cterm, nlag)13:  Extract normalized cointegrating vectors (evec)14:  **for** each cointegrating vector j = 1 to (rank(evec) − 1) **do**15:    **for** each signal k = 1 to *N* **do**16:    Compute residual *r_j_* = $\sum_{k}{signal}_{k}\times {evec}_{k,j}$
17:    Perform ADF test on residual *r_j_*; record corresponding ADF t-statistic18:   **end for**19:  **end for**20: **end for**

## 4. Experimental Work

This section reports experimental work undertaken to obtain vibration data used for misalignment detection. Firstly, the experimental test rig is described. Then, vibration data are illustrated in the time and frequency domains.

### 4.1. Rotor–Shaft Test Rig and Experimental Procedure

**Figure 2:**
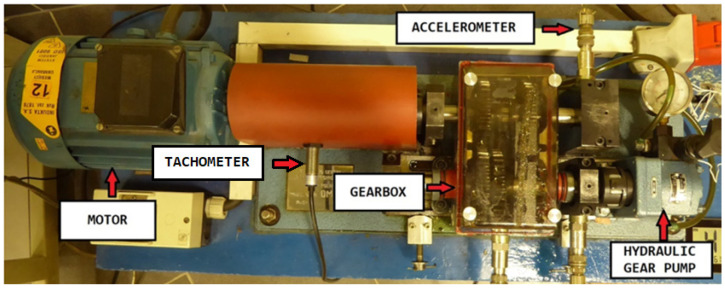
Experimental test rig used for misalignment fault detection.

The experimental setup for the rotor–shaft test rig, illustrated in Figure 2, consists of three primary components: an electric motor, a gearbox, and a hydraulic gear pump. The transmission system employs four identical spur gears arranged as two interacting pairs, each gear having 29 teeth. The gears are designed with a diametral pitch of 8 inches (20.32 cm), a tooth height of 6.16 mm, and a standard pressure angle of 20°. In this study, vibration measurements were obtained using an ICP 627A01 accelerometer, featuring a sensitivity of 10 mV/g, mounted on the bearing of the input gear. Shaft rotational speed was monitored with a SELS PCID-8ZN eddy-current tachometer. The analog vibration signals were digitized through a 24-bit multi-channel data acquisition system (VIBDaq 4+), ensuring high-resolution signal capture for further analysis. The collected vibration signals were processed using 10th-order Butterworth filters, applying both high-pass and low-pass configurations. A cutoff frequency of 7 Hz was set for the high-pass filter, while the low-pass filter cutoff was fixed at 4 kHz. Data acquisition was performed at a sampling rate of 11 kHz, with each recording containing 991,232 samples. Alongside vibration data, the oil temperature at the pump’s throttle valve was monitored, varying between 30.9 °C and 60.1 °C throughout the experiments. Since oil viscosity is temperature-dependent, these variations can alter the shaft’s rotational frequency. Moreover, as highlighted in [65], such effects may obscure or mask early fault signatures in rotor–shaft systems.

The experiments were designed to simulate different degrees of shaft misalignment under varying load conditions.

- Shaft misalignment was introduced by adjusting the separation between the two shafts of the gear transmission to 0, 0.25, 0.5, 0.75, and 1.0 mm. These increments produced progressive levels of parallel misalignment in the rotor–shaft system.
- For each misalignment setting, load variations were applied by regulating the pressure at the hydraulic gear pump’s throttle valve. The pressure was gradually increased from 1.6 MPa to 2.4 MPa in steps of 0.4 MPa, allowing for the assessment of misalignment effects under different loading conditions.

Vibration signals were collected under various shaft misalignment conditions. The rotational speed was derived from the tachometer measurements using the function “tachorpm” (MATLAB version R2019b), yielding an average speed of 1490.68 RPM (Revolutions Per Minute). This corresponds to roughly 442 samples per shaft revolution. From this, the shaft’s rotational frequency was calculated as 25 Hz, while the gear meshing frequency was determined to be 733 Hz.

It is noted that three accelerometers were installed on the experimental setup: one on the bearing of the input wheel, one on the gearbox cover, and one between the gearbox and the pump. The data presented in this paper was selected from the sensor on the bearing of the input wheel because, in the authors’ opinion, it provides the most representative vibration signal for misalignment detection. The sensor on the gearbox cover was not used for analysis due to the relatively low mechanical stiffness of the cover, which can lead to attenuated or noisy measurements. The sensor located between the gearbox and the pump was also not used because it might be influenced by additional mechanical interactions and transmission effects from the pump, which can obscure misalignment-related vibrations. The chosen sensor location therefore provides the most reliable and interpretable signal for the purpose of this study.

### 4.2. Vibration Data

**Figure 3:**
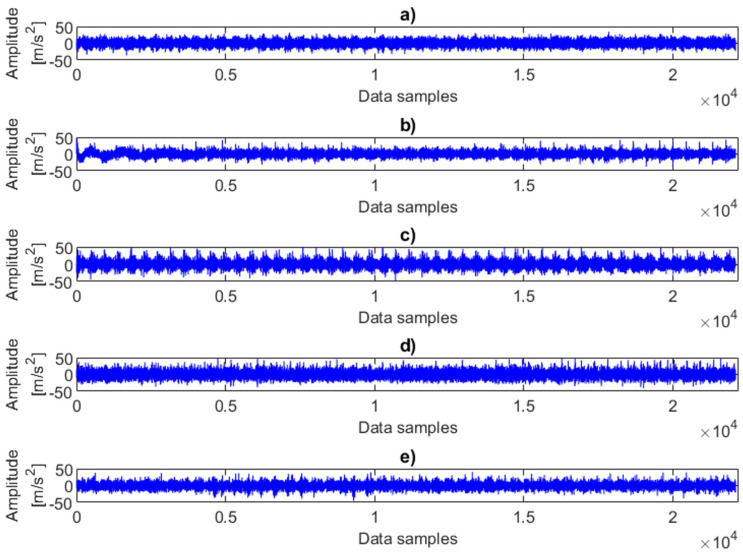
Examples of time-domain vibration data representing different combinations of faults and load conditions: (**a**) no fault with 2.4 MPa load, (**b**) 0.25 mm fault with 2 MPa fault, (**c**) 0.5 mm fault with 2 MPa fault, (**d**) 0.75 mm fault with 1.6 MPa fault, (**e**) 1 mm fault with 0 MPa (no load).

Figure 3 presents examples of time-domain vibration data under various combinations of faults and load conditions. Although the signals were recorded from five different operating states, the plots reveal similar patterns and comparable amplitudes. In other words, the variations in fault severity are not clearly distinguishable, as the effects of changing load strongly dominate the vibration response. Table 1, Table 2 and Table 3 demonstrate that the statistical parameters derived from raw vibration signals are significantly dependent on the load applied to the system. Even under identical fault conditions, the influence of load is clearly observable, leading to the conclusion that it is not feasible to detect misalignment in systems operating under varying operational conditions based solely on raw vibration analysis.

**Figure 4:**
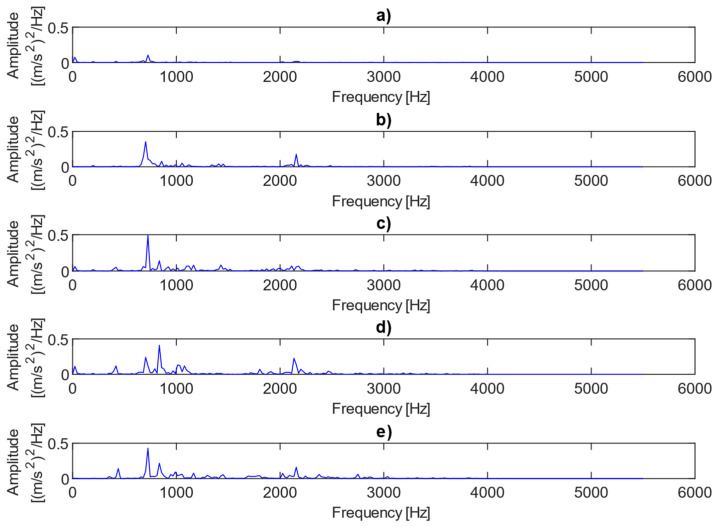
Power spectra for vibration data representing different fault conditions with the presence of no load: (**a**) no fault, (**b**) 0.25 mm fault, (**c**) 0.5 mm fault, (**d**) 0.75 mm fault, (**e**) 1 mm fault.

**Figure 5:**
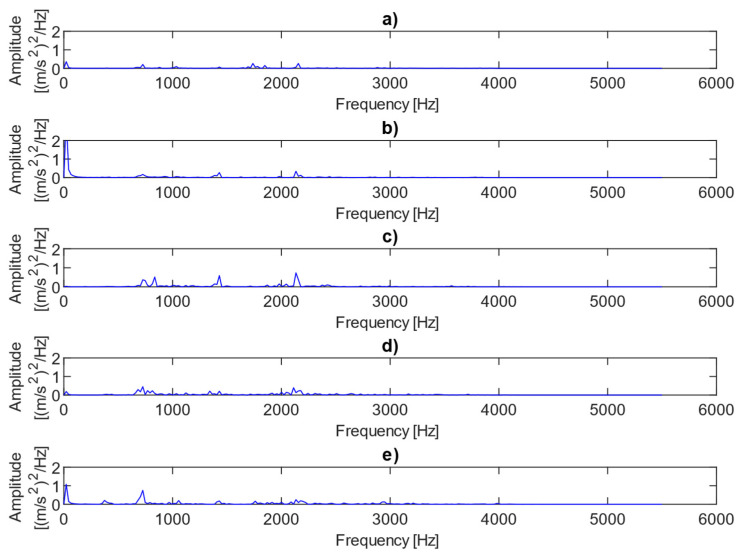
Power spectra for vibration data representing different fault conditions with the presence of a 2 MPa load: (**a**) no fault, (**b**) 0.25 mm fault, (**c**) 0.5 mm fault, (**d**) 0.75 mm fault, (**e**) 1 mm fault.

Figure 4 shows examples of power spectra for the vibration data representing different fault conditions under the presence of no load. The results clearly show three dominant spectral components, i.e., the first harmonic of the rotational frequency (25 Hz), and the first (733 Hz) and third (2143 Hz) harmonics of the meshing frequency. The first harmonic (733 Hz) is only visible in cases where there is 0.25 mm or 0.50 mm fault. As the level of misalignment increases, the height and number of sidebands also increase, as illustrated in Figure 4. The power spectra of the vibration data for different fault conditions under the presence of a 2 MPa load are plotted in Figure 5. In this case, the amplitudes of the meshing harmonics vary across the different misalignment conditions, providing little clear evidence of either the presence or progression of misalignment.

In summary, the analysis of time- and frequency-domain vibration data is not easy for the detection of misalignment.

## 5. Cointegration-Based Approach for Misalignment Detection

### 5.1. Terminology for Data Description and Analysis

For clarity in analyzing and interpreting the misalignment detection results, this study adopts a set of notations to represent different fault severities and load conditions:

- no fault—indicates no separation between the two shafts of the gear transmission;
- 0.25 mm fault—indicates a 0.25 mm separation between the two shafts;
- 0.5 mm fault—indicates a 0.5 mm separation between the two shafts;
- 0.75 mm fault—indicates a 0.75 mm separation between the two shafts;
- 1 mm fault—indicates a 1 mm separation between the two shafts;
- *N* MPa load—represents a throttle valve pressure of *N* MPa in the gear pump, where *N* = 1.6, 2.0, or 2.4.

In addition, terminology related to the cointegration process is defined as follows:

- Pre-cointegrated data—refers to vibration measurements prior to applying cointegration;
- Post-cointegrated data—refers to vibration measurements after the application of cointegration.

### 5.2. Cointegration Procedure and Parameters Used

The vibration data were analyzed using a combination of cointegration analysis and the ADF test. The integrated procedure comprised three main schemes:

1. Conducting the ADF test on the pre-cointegrated data;
2. Applying cointegration to the vibration signals;
3. Performing the ADF test on the post-cointegrated data.

The ADF test serves as an indicator of a signal’s stationarity. Generally, a more negative ADF t-statistic implies a higher degree of stationarity [56,61]. The assumption is that vibration measurements are stationary I(0) time series. Nevertheless, introducing misalignment into the system can disrupt this stationarity. Moreover, varying degrees of misalignment may cause differing stationarity patterns in the vibration signals. If such variation is observed, it becomes possible not only to identify the presence of misalignment but also to evaluate its intensity. This concept forms the basis of the methodology adopted in this study.

When applying the ADF test [57] and Johansen’s cointegration procedure [63], it is important to select suitable approximation models, which are the test regression models and the VECM, such that they are appropriate to the SHM data under consideration. In addition, the lag length values used in these approximation models need to be properly selected so that the error is a white noise process. In this work, the lag length was determined using the stationarity-based selection method described in [61]. Based on this approach, a value of *p* = 15 was chosen and applied consistently to both the ADF test and the cointegration analysis.

## 6. Misalignment Detection Results

In preparation for the unit root and cointegration analysis, vibration data obtained from each measurement instance were segmented into ten separate time series, each consisting of 22,100 samples. This segmentation approach provides multiple advantages when dealing with stationarity testing, particularly with the ADF test. By analyzing shorter and distinct segments instead of a single long signal, the likelihood of capturing non-stationary fluctuations diminishes, while local stationarity patterns become more visible. Furthermore, this approach improves the statistical robustness of the cointegration analysis, as each segment can be treated as an independent realization of the underlying stochastic process, enabling cross-comparison between different misalignment states under varying loads.

### 6.1. Conducting the ADF Test on the Pre-Cointegrated Data

This scheme can be illustrated through the following sequence:

Vibration data=>ADF tests=>t−statistics

The above scheme means that ADF tests were applied to the segmented vibration signals for three different simulated load levels: 1.6 MPa, 2.0 MPa, and 2.4 MPa. For each load case, data was obtained under five misalignment conditions: no fault, 0.25 mm fault, 0.5 mm fault, 0.75 mm fault, and 1 mm fault. The test was performed individually for each time series segment, and the corresponding t-statistics were recorded to evaluate the level of stationarity.

The analysis revealed that in each load scenario, the signals corresponding to the no fault case generally displayed more negative t-statistics, suggesting a higher degree of stationarity. This observation indicates that the ADF test may be capable of identifying the no fault condition based solely on the statistical properties of the vibration data. However, the separation between the no fault and other conditions was not substantial, with overlapping t-statistics observed across the remaining misalignment levels.

**Figure 6:**
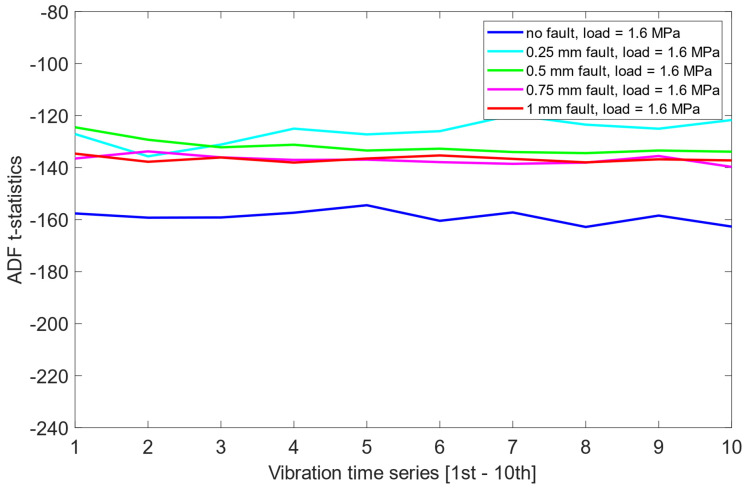
ADF test results on the pre-cointegrated data with the presence of a 1.6 MPa load.

**Figure 7:**
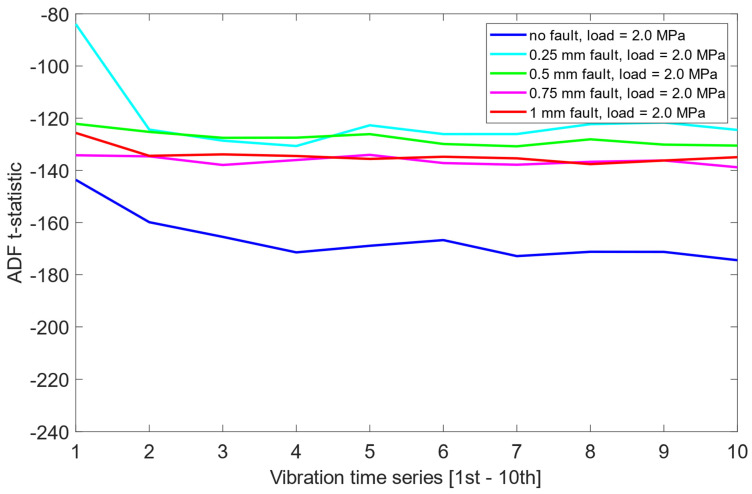
ADF test results on the pre-cointegrated data with the presence of a 2.0 MPa load.

**Figure 8:**
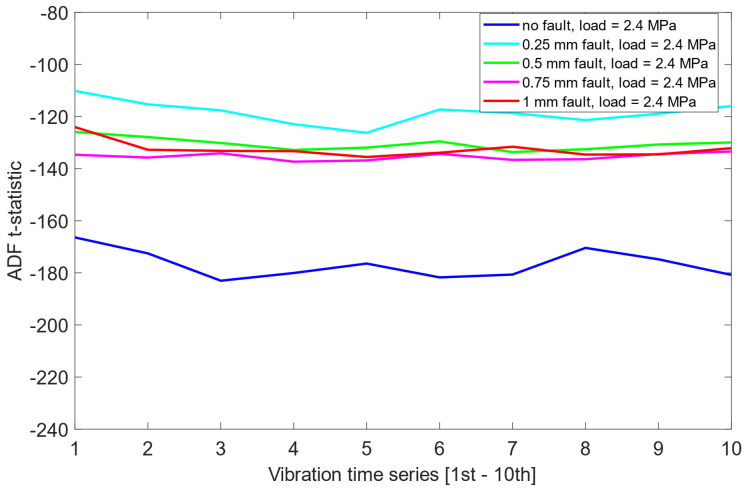
ADF test results on the pre-cointegrated data with the presence of a 2.4 MPa load.

Furthermore, the distribution of t-statistics among the faulty states did not exhibit any consistent or interpretable pattern across load cases. As presented in Figure 6, Figure 7 and Figure 8, this suggests that while the ADF test offers some limited sensitivity to the presence of misalignment, it fails to provide reliable information about the severity of the fault.

### 6.2. Applying Cointegration to the Vibration Signals

This scheme can be explained through the following sequence:

Vibration data=>cointegration=>cointegration residuals

As presented in the above scheme, following the initial ADF analysis, cointegration analysis was conducted for each of the three load cases using the full set of misalignment conditions. Johansen’s cointegration procedure was used in this scheme. The objective was to extract cointegration residuals that may suppress shared trends or components across the data, potentially improving the sensitivity of fault detection in subsequent statistical testing.

**Figure 9:**
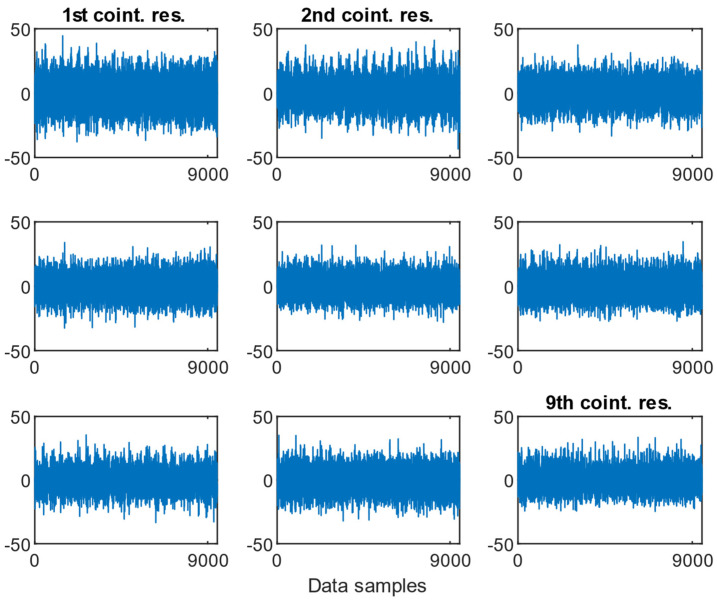
Examples of the cointegration residuals for the case of a 0.5 mm fault with the presence of a 1.6 MPa load.

**Figure 10:**
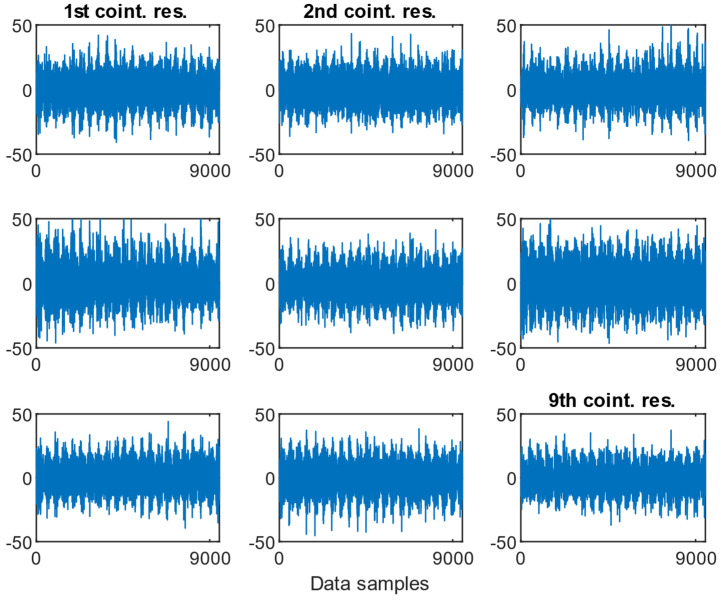
Examples of the cointegration residuals for the case: 0.5 mm fault with the presence of a 2.0 MPa load.

**Figure 11:**
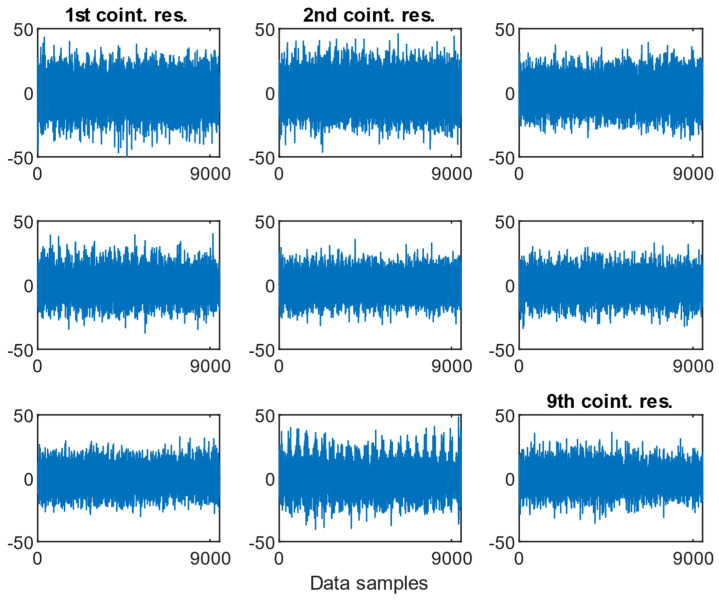
Examples of the cointegration residuals for the case: 0.5 mm fault with the presence of a 2.4 MPa load.

As presented in Figure 9, Figure 10 and Figure 11, the resulting cointegration residuals, however, did not exhibit any visually consistent structure or pattern when plotted across the different misalignment states. No clear trend or change in variability could be identified that would allow for direct detection or classification of misalignment severity. Instead, these residuals served as intermediate transformed signals for further testing, and they were used as inputs for the ADF test applied to the post-cointegrated data.

### 6.3. Performing the ADF Test on the Post-Cointegrated Data

This scheme is described by the following sequence:

Vibration data=>cointegration=>ADF tests=>t−statistics

The cointegration residuals for each of the three load levels (1.6 MPa, 2.0 MPa, 2.4 MPa) were subjected to the same ADF testing methodology as in Section 6.1. Again, each residual signal corresponded to one of five misalignment states, and the test was repeated for all segmented time series.

**Figure 12:**
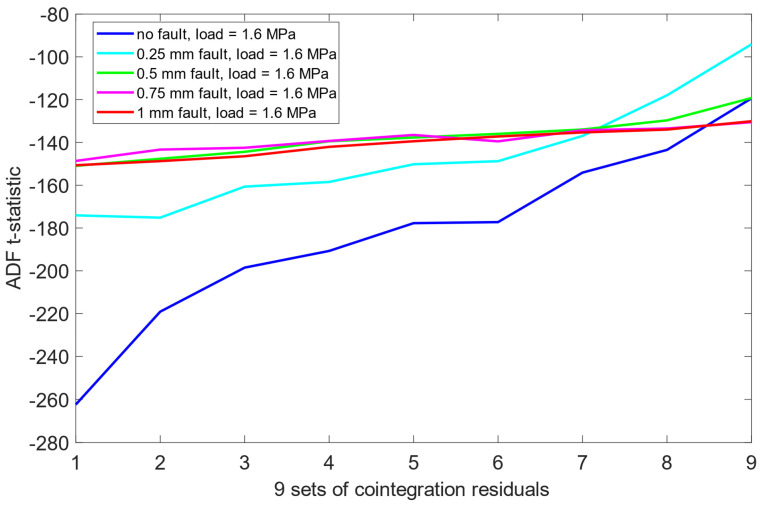
ADF test results on the post-cointegrated data with the presence of a 1.6 MPa load.

**Figure 13:**
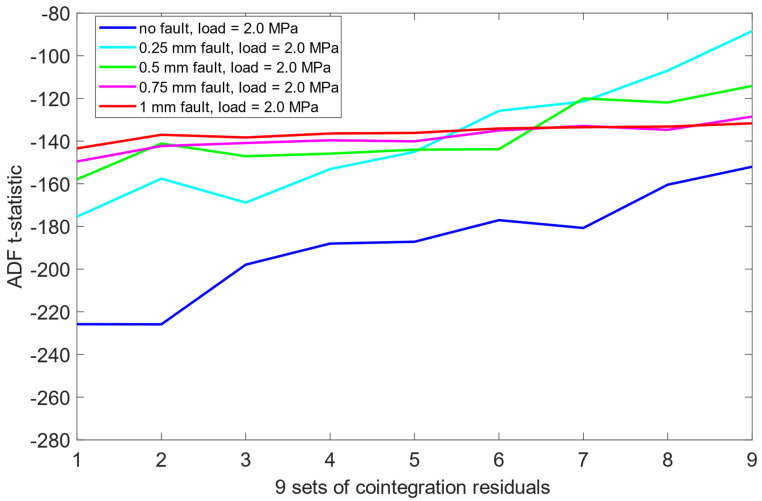
ADF test results on the post-cointegrated data with the presence of a 2.0 MPa load.

**Figure 14:**
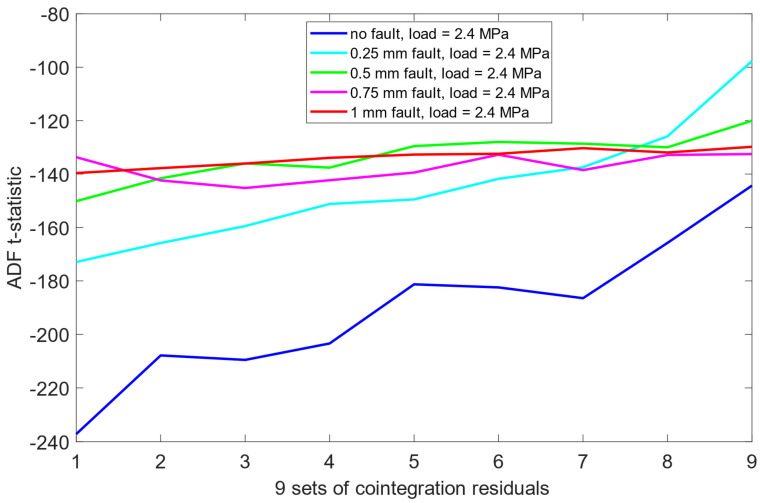
ADF test results on the post-cointegrated data with the presence of a 2.4 MPa load.

Unlike the results obtained previously for the pre-cointegration data case in Section 6.1, the ADF test results for this scheme revealed significantly improved separability between the no fault condition (0 mm misalignment) and the remaining misalignment cases. For each load scenario, ADF t-statistics for the 0 mm signals were consistently and clearly more negative than those for faulty states. This indicates that cointegration analysis enhanced the sensitivity of the ADF test to misalignment presence. Moreover, an interesting structural pattern emerged: for signals representing healthy state or small misalignment levels (e.g., 0.25 mm), the behavior of the ADF t-statistics began to resemble fractal-like variability—a phenomenon more pronounced as the severity of the misalignment decreased. As presented in Figure 12, Figure 13 and Figure 14, this observation was consistent across all three load scenarios.

A comparative summary of ADF t-statistics separation between misalignment states, both before and after cointegration, presented in Table 4 and Table 5, indicates that the combination of cointegration and ADF testing provides detection capability when compared to raw vibration data alone. The improved separation of the no-fault state and the development of structured patterns in ADF t-statistics across misalignment severities suggest that this methodology may serve as a reliable tool in automated misalignment detection. Given its sensitivity and robustness across varying load conditions, the approach holds promise for early-stage fault identification in rotating machinery systems.

Importantly, the cointegration process was effective in suppressing the influence of varying load levels, allowing the ADF test to detect misalignment characteristics independently of the applied load. This invariance to load conditions further reinforces the robustness and practical applicability of the proposed approach in real-world operating environments.

### 6.4. Discussion

While modern machine learning (ML) approaches such as CNNs, autoencoders, GANs, and Transformers have been widely applied to fault diagnosis under varying or non-stationary conditions, the cointegration-based method proposed in this study offers several complementary advantages:

- Principled handling of non-stationarity: Cointegration explicitly models long-run equilibrium relationships between non-stationary signals (e.g., vibration vs. load), detecting departures that indicate faults. Unlike many ML methods, it does not require exhaustive coverage of operating regimes to handle varying load.
- Low data and label requirements: The method operates primarily in an unsupervised manner, learning healthy system relationships and detecting anomalies without requiring extensive labeled fault data.
- Robustness and low computational cost: Cointegration estimation is computationally lightweight, less prone to overfitting, and suitable for real-time or edge deployment compared with large ML models.
- Statistical rigor: Detection thresholds can be set based on hypothesis testing of residual stationarity, providing explicit confidence levels for alarms.
- Complementarity with ML: Cointegration features can feed ML models or be part of hybrid systems, combining interpretability with nonlinear feature extraction when large labeled datasets are available.

The authors are also aware of the limitations of the cointegration method relative to ML approaches. Standard cointegration is linear and may underperform when faults manifest through complex nonlinear interactions or high-dimensional sensor arrays, where ML methods typically excel.

## 7. Conclusions

This study introduced a cointegration-based framework for vibration-based misalignment detection in rotating machinery operating under varying load conditions. The approach integrates cointegration analysis with the ADF test to examine differences in the stationarity characteristics of vibration signals before and after cointegration. The methodology was implemented through three testing schemes: (1) applying the ADF test directly to the raw vibration data, (2) performing cointegration on the vibration series, and (3) applying the ADF test to the post-cointegration data.

The key findings can be summarized as follows:

- Applying the ADF test to the raw (pre-cointegrated) data allows for detection of the healthy condition; however, fault severity cannot be reliably distinguished since misalignment states show no clear structural patterns.
- Cointegration residuals do not display distinct drifts, trends, or variability changes that would enable direct identification or classification of misalignment severity.
- When the ADF test is applied to post-cointegrated data, the separation between healthy and faulty conditions becomes more evident. Furthermore, for signals corresponding to the healthy state or minor misalignments (e.g., 0.25 mm), the ADF t-statistics reveal a fractal-like variability pattern, which becomes more pronounced as misalignment severity decreases.

Although the findings demonstrate the potential of the proposed method, further research is necessary to validate and extend the approach as well as to compare it with current advanced fault diagnosis methods. Future work should investigate more complex datasets, a broader range of operating and environmental conditions, and additional load scenarios. In addition, the present work can be extended by exploring hybrid approaches that combine cointegration with machine learning methods. This line of work aims to leverage the interpretability and statistical rigor of cointegration while benefiting from the nonlinear feature extraction and high-dimensional pattern recognition capabilities of ML models. Moreover, further exploration of the proposed methodology is required to enhance its robustness and applicability for practical damage detection.

## Figures and Tables

**Table 1:** Statistical characteristics of vibration responses recorded at different misalignment conditions under 1.6 MPa load.

Parameter	No Fault Condition	0.25 mm Fault Condition	0.5 mm Fault Condition	0.75 mm Fault Condition	1 mm Fault Condition
Peak-to-peak amplitude (m/s^2^)	55.01	87.8	111.33	122.7	149.66
Mean value (m/s^2^)	0	0	0	0	0
RMS value (m/s^2^)	7	8.5	10	11.8	12.6

**Table 2:** Statistical characteristics of vibration responses recorded at different misalignment conditions under 2.0 MPa load.

Parameter	No Fault Condition	0.25 mm Fault Condition	0.5 mm Fault Condition	0.75 mm Fault Condition	1 mm Fault Condition
Peak-to-peak amplitude (m/s^2^)	68.72	105.01	125.57	139.24	153.84
Mean value (m/s^2^)	0	0	0	0	0
RMS value (m/s^2^)	7.96	9	11.24	12.74	13.18

**Table 3:** Statistical characteristics of vibration responses recorded at different misalignment conditions under 2.4 MPa load.

Parameter	No Fault Condition	0.25 mm Fault Condition	0.5 mm Fault Condition	0.75 mm Fault Condition	1 mm Fault Condition
Peak-to-peak amplitude (m/s^2^)	79.34	111.78	121.95	121.49	148.53
Mean value (m/s^2^)	0	0	0	0	0
RMS value (m/s^2^)	9	9.79	12.44	13.47	14.31

**Table 4:** Comparison of ADF t-statistics for no fault, 0.25 mm fault, and 0.5mm fault cases.

	No Fault Condition			0.25 mm Fault Condition			0.5 mm Fault Condition		
Load [MPa]	1.6	2.0	2.4	1.6	2.0	2.4	1.6	2.0	2.4
Average t-statistic from vibration data	−158.959	−166.583	−176.685	−126.225	−121.093	−118.464	−131.919	−127.793	−130.517
Separation relative to no fault	—	—	—	32.7345	45.49	58.221	27.04	38.79	46.168
Average t-statistic from cointegration residuals	−182.528	−188.345	−190.929	−146.271	−138.08	−144.627	−137.672	−137.326	−133.496
Relative to no fault	—	—	—	36.2575	50.265	46.301	44.856	51.019	57.433

**Table 5:** Comparison of ADF t-statistics for 0.75 mm fault and 1mm fault cases.

	0.75 mm Fault Condition			1 mm Fault Condition		
Load [MPa]	1.6	2.0	1.6	2.0	1.6	2.0
Average t-statistic from vibration data	−137.022	−136.366	−137.022	−136.366	−137.022	−136.366
Separation relative to no fault	21.9376	30.2169	21.9376	30.2169	21.9376	30.2169
Average t-statistic from cointegration residuals	−138.683	−138.184	−138.683	−138.184	−138.683	−138.184
Relative to no fault	—	—	—	36.2575	50.265	46.301

## Data Availability

The original contributions presented in this study are included in the article. Further inquiries can be directed to the corresponding author.
